# The effect of enteral and parenteral feeding on secretion of orexigenic peptides in infants

**DOI:** 10.1186/1471-230X-9-92

**Published:** 2009-12-10

**Authors:** Przemyslaw J Tomasik, Krystyna Sztefko

**Affiliations:** 1 Department of Clinical Biochemistry, Polish-American Children's Hospital, Jagiellonian University, Wielicka St 265, 30-663 Krakow, Poland

## Abstract

**Background:**

The feeding in the first months of the life seems to influence the risks of obesity and affinity to some diseases including atherosclerosis. The mechanisms of these relations are unknown, however, the modification of hormonal action can likely be taken into account. Therefore, in this study the levels of ghrelin and orexin A - peripheral and central peptide from the orexigenic gut-brain axis were determined.

**Methods:**

Fasting and one hour after the meal plasma concentrations of ghrelin and orexin were measured in breast-fed (group I; n = 17), milk formula-fed (group II; n = 16) and highly hydrolyzed, hypoallergic formula-fed (group III; n = 14) groups, age matched infants (mean 4 months) as well as in children with iv provision of nutrients (glucose - group IV; n = 15; total parenteral nutrition - group V; n = 14). Peptides were determined using EIA commercial kits.

**Results:**

Despite the similar caloric intake in orally fed children the fasting ghrelin and orexin levels were significantly lower in the breast-fed children (0.37 ± 0.17 and 1.24 ± 0.29 ng/ml, respectively) than in the remaining groups (0.5 ± 0.27 and 1.64 ± 0.52 ng/ml, respectively in group II and 0.77 ± 0.27 and 2.04 ± 1.1 ng/ml, respectively, in group III). The postprandial concentrations of ghrelin increased to 0.87 ± 0.29 ng/ml, p < 0.002 and 0.76 ± 0.26 ng/ml, p < 0.01 in groups I and II, respectively as compared to fasting values. The decrease in concentration of ghrelin after the meal was observed only in group III (0.47 ± 0.24 ng/ml). The feeding did not influence the orexin concentration. In groups IV and V the ghrelin and orexin levels resembled those in milk formula-fed children.

**Conclusion:**

The highly hydrolyzed diet strongly affects fasting and postprandial ghrelin and orexin plasma concentrations with possible negative effect on short- and long-time effects on development. Also total parenteral nutrition with the continuous stimulation and lack of fasting/postprandial modulation might be responsible for disturbed development in children fed this way.

## Background

The human milk is superior baby nutrient. Mother milk has the right content of fat, sugar, water, and protein required for a baby growth and development. An advantage of the human milk over milk formulas is commonly known. However, the milk formulas, products chemically resembling the natural human milk are sometimes in use. Under certain circumstances, the enteral feeding is impossible and all water, energy and body structure materials are delivered directly to the vessels. In shorter periods of the deprivation of enteral feeding, organism may be intravenously supplemented with the saline and glucose solutions, but in longer periods of food deprivation, the parenteral nutrition is required. The short-time and long-time consequences of enteral and parenteral feeding are not clear, especially for growing organisms.

Discovery of gastrointestinal peptides which influence the central regulation of the food intake (directly or indirectly via vagus nerve) provided establishing of a two-way connection termed gut-brain axis [1]. Peripheral ghrelin and mainly central orexins are involved in the orexigenic part of this axis. Both these peptides stimulate the expression and releasing of neuropeptide Y (NPY) - the central neuromediator in feeding behavior [2].

Ghrelin is a 28-amino acid peptide produced principally in the stomach and small intestine [3]. Initially, ghrelin was considered responsible for a growth hormone release [4]. At the present, its role in the formation of eating behavior and weight regulation was recognized. Ghrelin is up-regulated in fasting, plasma ghrelin peaks before a regular meal, then progressively decreases. Its increase to another peak value just before the next meal suggests that ghrelin might act as a meal initiator [5]. Ghrelin, known mainly as a short-time regulator, is also involved in a long-term regulation. In respect to normal-weight individuals, circulating levels of ghrelin are lower in obese and elevated in anorectic patients. The levels of this peptide negatively correlated with body mass index [6,7]. The increase in ghrelin during starvation boosts eating behavior, induces body weight gain and adiposity through stimulating food intake and reducing fat utilization or energy expenditure [8]. Intracerebraventricular administration of ghrelin stimulates both growth hormone secretion and food intake [9,10].

Orexins A (OXA) and B, 33 and 28 amino acid peptides, respectively, originate from 130 amino acid preproorexin produced by neurons located in the hypothalamus and in the spinal cord [10-12]. However, neurons and endocrine cells in the gut and pancreas also exhibit orexin-like immunoreactivity [13]. The projection of central orexin nerves in humans leads to locus coeruleus, dorsal raphe nuclei, amygdala, suprachismatic nucleus, basal forebrain, cholinergic brainstem and spinal cord [14]. Orexins are involved in developing behavior patterns controlled by the hypothalamic region [15]. The Greek origin name of these peptides clearly reflects basic function of orexins (orexigenic peptides) - orexos means "to eat" - i. e. positive stimulation of energy balance. Similarly to ghrelin, a single intracerebroventricular injection of OXA increased caloric intake when administered during the light phase but not at the beginning of the dark phase, indicating that sensitivity to orexins might be controlled by circadian variations [16]. Orexin neurons interact with hypothalamic feeding pathways and monoaminergic/cholinergic centers, thus probably, linking energy balance with sleep/wakefulness phases and they motivate food seeking [17]. The studies revealed that orexins cooperate also in regulation of blood pressure and heart rate and release of some hypothalamic hormones [18].

Ghrelin and orexins with some other peptides constitute jointly an orexigenic part of gut-brain axis which integrates and regulates the homeostasis of energy balance and feeding [19]. Ghrelin stimulates feeding through NPY and AgRP, orexigenic peptides co-localized in neurons of the hypothalamic arcuate nucleus [20]. NPY fibers directly project to orexin neurons [21]. Intracerebroventricular injection of anti-orexin antiserum prior to the NPY injection significantly attenuated NPY-induced feeding, indicating that NPY interacts with orexins anatomically and functionally [22]. There are suggestions that ghrelin partly employs vagal afferent loops [23]. The next, central messengers, orexins A and B, are stimulated also with glucose.

Type of feeding within first months of the life has a great impact upon obesity in the subsequent life periods and can produce even long-life effects [24]. Breast-feeding of newborns and infants improves lipid profile and lowers blood pressure in later life and decreases the risk of developing obesity [25-28]. Likely, the way of feeding alters the gut-brain axis and generates short and long term differences in the infant development. Also a deprivation of enteral feeding by substituting it with a parenteral nutrition may negatively influence the gut-brain axis. Although orexins and ghrelin were discovered over 10 years ago, no data are available on the influence of the diet used in the infants' nutrition upon the secretion of ghrelin and OXA. The bypassing of the gut which should result in the strong disturbances in the secretion of hormones involved in the gut-brain axis also remains unrecognized. Fasting and postprandial plasma levels of ghrelin and OXA in nursed infants, formula-fed infants, babies maintained on a hypoallergic highly hydrolyzed diet or children on total parenteral nutrition were determined in this study.

## Methods

### Experimental groups

The enterally fed children were divided into the following groups: breast-fed infants (group I; n = 17, 10 girls and 7 boys) and cow milk formula-fed infants i.e. with Bebilon 1, Nutricia - formula for younger infants, equal to Nutrilon 1; (group II; n = 16, 9 girls and 7 boys). All these children were healthy. Group III (n = 14, 7 girls and 7 boys) consisted of infants allergic to milk protein and, therefore, fed with the highly hydrolyzed milk protein formula diet (Nutramigen; Bristol Myers Squibb & Mead Johnson). The diagnosis of cow milk allergy (CMA) was issued based on typical symptoms observed on former physical examinations. The plasma levels of specific anti-CMA IgE antibodies determined for a majority of children from group III were pathologically elevated, although only the children without physical symptoms of allergy at least within two weeks before the study, were qualified to the study. In all these children the diet remained unchanged for at least two weeks before the test, and in group III that period lasted at least 4 weeks. The children were of similar age (group I - 3.5 ± 1.5 months; group II - 4 ± 1 month, group III - 4 ± 1.5 months). Mean body mass was appropriate to the age and length (group I - 6.2 ± 0.9 kg; group II - 5.5 ± 0.8 kg, group III -5.6 ± 1.0 kg). Coexistence of other diseases and any hereditary connections were excluded in all the subjects.

Children from group I received breast at any signs of hunger - they were nursed 7-8 times a day. Children from groups II and III received bottle usually 7 times a day also when they showed signs of hunger. In all cases the diet did not change for at least 2 weeks prior to the blood test sampling. The calorie intake was adequate to the children age and body weight. All infants were fed exclusively with either milk or formula free of solids or mixed solids.

The children in group IV (n = 15, 6 girls and 9 boys) were prepared for computed tomography in sedation and, therefore, they were deprived of food for at least 8 hours prior to the imaging. In this period they received fluids (0.9% saline and 5% glucose mixture 2:1 vv) in the volume following their specific requirements for fluids. Finally recruited children were free of pathologies. The group V (n = 14, 6 girls and 8 boys) included infants requiring a total parenteral nutrition for either congenital atresia or acquired obstruction of gastrointestinal tract. The children with total parenteral nutrition fully met their amino acid requirements (Vaminolact, Fresenius Kabi). The level of administered non-protein calories i.e. 70% glucose, 30% lipid emulsion (Intralipid) was calculated based on the amount of infused amino acids (175 kcal of non-proteins per 1 gram of amino acid nitrogen).

### Protocol of the study

The Permanent Ethical Committee for Clinical Studies of the Medical College of the Jagiellonian University has approved the protocol of the study, and the parents of examined children provided their signed consent. Before feeding, naked children were examined and weighted with an electronic integrating balance. The crown-to-heel length was measured with the 1 mm precision on a recumbent infant board. Percentage of ideal body weight was calculated using adequate data for the Polish population [29].

The catheter was fixed in vein of each child and venous blood was taken directly prior to the meal. Subsequently, children consumed a portion of either milk or formula, and the blood samples were collected 60 minutes after this stimulation. For all groups, the mean volume of either milk or milk formulas ingested per day was 620 ± 120 ml. The consumption lasted no longer than 30 minutes (mean 16 min). In children with total parenteral nutrition the blood was taken once, after minimum 24 h continuous infusion. In children with intravenous glucose infusion, blood was collected also once after minimum 8 h continuous infusion.

Blood samples for the hormone determination were collected into chilled glass tubes with 4 mg EDTA and 0.2 TIU (trypsin inhibitor unit) of aprotinin (Sigma, USA). Immediately after the sampling, the tubes were transported to the laboratory in an icebox. Blood was centrifuged for 10 min at 3000 g and +4°C. Plasma was stored at -80°C until the measurements. Ghrelin (total) as well as OXA were determined with the EIA method (Phoenix Pharmaceuticals, Inc, USA). The glucose levels were determined in serum using method with glucose oxidase.

In all estimations the intraassay coefficient of variation was below 5%. The OXA assay exhibited 18% cross-reactivity with orexin B and no cross-reactivity with other substances, such as NPY, MSH, leptin or CRP. Sensitivity of the OXA assays was 0.06 ng/ml. The ghrelin kit had no cross-reactivity with any other peptides. This method offered the minimum detectable concentration of ghrelin as low as 0.08 ng/ml.

Differences between groups were tested for statistical significance involving one-way analysis of variance followed by the Bonferroni multiple comparison procedure or, when appropriate, by Student's t-test (paired when compared parameters before and after feeding in the same group). Parametric and non-parametric Spearman rank correlations were used to evaluate relations between different variables. P ≤ 0.05 was considered significant.

## Results

There were no statistical differences in the age and body weight between studied groups (table 1). For the breast-fed children uptake of 108 kcal/kg/24 h was accepted as recommended value of daily energy intake for 4 month children in the Guideline of the Polish National Food and Nutrition Institute. The consumption of energy in groups II and III was calculated from the volume of ingested formulas within 24 hours, taking into account the body weight and energetic value of consumed milks reported by the manufacturers. The children fed with primary formula based on cow milk consumed mainly 108 ± 18 kcal/kg/24 h, and children fed with highly hydrolyzed protein formula consumed 108 ± 22 kcal/kg/24 h. The calorie intake in these groups was almost like one another and significantly higher than in children fed parenterally (p < 0.001). Children with TPN received 60 ± 8 kcal/kg/24 h, whereas children maintained on iv saline/glucose mixture received only 37 ± 6 kcal/kg/24 h.

**Table 1:** Clinical characteristics of studied children groups expressed as mean ± SD.

Group		Food	Age (months ± SD)	Body weight (kg ± SD)
Number	Population (n)			
I	17	Human milk	3.5 ± 1.5	6.2 ± 0.9

II	16	Cow milk primary formula (Bebilon 1)	4 ± 1	5.5 ± 0.8

III	14	Highly hydrolyzed protein formula (Nutramigen)	4 ± 2	5.6 ± 1.0

IV	15	Infusion of glucose/saline mixture	4.5 ± 1.5	6.3 ± 1.9

V	14	Total parenteral nutrition	4 ± 2	4.9 ± 1.8

**Figure 1:**
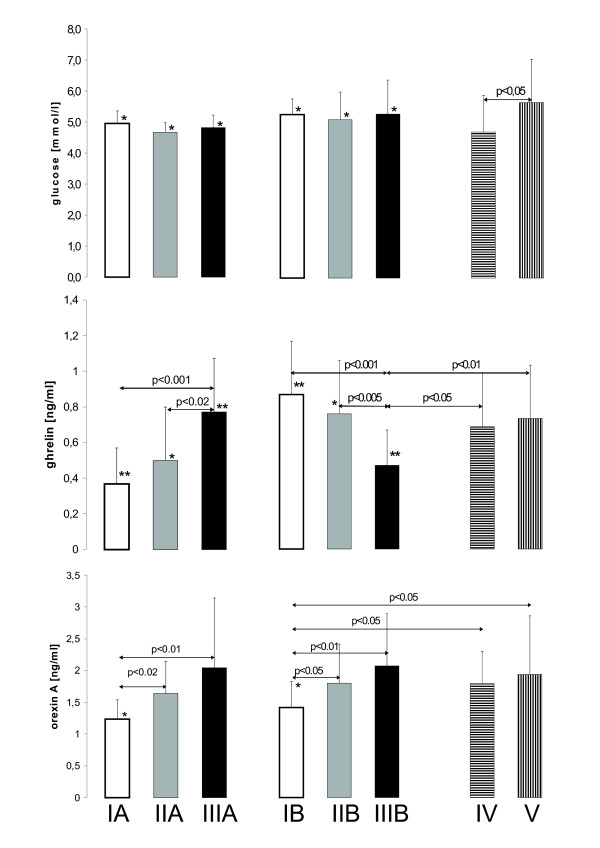
**The mean concentrations of ghrelin, orexin and glucose**. The levels of determined parameters before meal (groups IA, IIA, IIIA), one hour after the meal (groups IB, IIB, IIIB) and in parenterally fed groups IV and V. The differences in one parameter between the same group before and after the meal (paired t-test) are marked with asterisks (* = p < 0.05; ** = p < 0.001).

There were no differences in the fasting glucose levels (group I - 5.0 ± 0.4 mmol/l, II - 4.7 ± 0.3 mmol/l and III - 4.8 ± 0.4 mmol/l). Also one hour after the meal there were no differences between groups in the glucose concentration (I - 5.2 ± 0.5 mmol/l; II - 5.1 ± 0.8 mmol/l; III - 5.5 ± 1.1 mmol/l) (figure 1), however in every group postprandial glucose levels were significantly higher than fasting levels (Student's paired test). In children with intravenous infusion of glucose and crystalloids and on the total parenteral nutrition, the mean glucose levels were similar (4.7 ± 1.2 mmol/l and 5.7 ± 1.4 mmol/l, respectively).

Concentration of ghrelin in group I before the meal was lower (0.37 ± 0.17 ng/ml) than in groups II (0.5 ± 0.27 ng/ml) and III (0.77 ± 0.27 ng/ml; p < 0.005). It is interesting, that in groups I and II within 1 hr after feeding, ghrelin increased over the fasting value to 0.87 ± 0.29 ng/ml, p < 0.002 and 0.76 ± 0.26 ng/ml, p < 0.01, respectively. The decrease in the concentration of ghrelin after the meal was observed only in group III (0.47 ± 0.24 ng/ml). In groups IV and V, the ghrelin levels were similar (0.69 ± 0.3 ng/ml and 0.73 ± 0.3 ng/ml, respectively) and resembled these observed in nursed children (figure 1).

The mean values of the OXA level in group I before (1.24 ± 0.29 ng/ml) and 1 hr. after the meal (1.38 ± 0.4 ng/ml) were significantly lower (p < 0.02) than the mean OXA concentrations in group II (1.64 ± 0.52 ng/ml and 1.76 ± 0.59 ng/ml, respectively) and in group III (2.04 ± 1.1 ng/ml and 2.02 ± 0.8 ng/ml, respectively). In groups IV and V, the OXA levels were similar (1.75 ± 0.53 ng/ml and 1.89 ± 0.89 ng/ml, respectively) and resembled these observed in the milk formula fed children (figure 1)

### Correlations

There were no significant correlations between analyzed parameters employing non-parametric Spearman test in all studied children. However, in subsequent groups a significant correlations were found between glucose and ghrelin. They were positive in children fed with milk and negative in children fed with highly hydrolyzed formula (table 2).

**Table 2:** Correlations coefficients between studied parameters in particular groups.

Group	Breast-fed	Cow milk formula	Highly hydrolyzed formula	Glucose-saline iv	Total parenteral nutrition
Glucose/OXA	0.07	-0.48 *	-0.02	-0.02	0.21

Glucose/ghrelin	0.47 *	0.32 *	-0.43 *	-0.16	0.29

OXA/ghrelin	0.18	0.12	-0.49 *	-0.03	-0.21

* p < 0.05

## Discussion

Several experiments showed that ghrelin and orexins stimulate hyperphagia and they strongly participated in the short-term as well as in the long-term energy balance [30-33]. Up to date, few papers analyzed the influence of the type of feeding on the fasting ghrelin outcome in few months period [34-36]. The direct effect of different formulas on fasting and postprandial ghrelin as well as analysis of ghrelin together with OXA as a part of gut-brain axis have not been reported. In this study, the response of glucose, ghrelin and OXA to a single oral bolus of formulas used in the infants' nutrition, as well as in the parenterally fed babies has been investigated.

### Orally fed children

Fasting glucose in orally fed groups was almost the same. One hour after the feeding, the blood glucose levels were higher than on fasting and its levels were similar in all studied groups. In spite of these similarities, the significant differences were noted in the fasting and postprandial plasma concentrations of ghrelin and orexin. The fasting ghrelin and orexin levels were the lowest in the breast-fed children, and the highest levels of those peptides were observed in the children fed with highly hydrolyzed diet. Similar results reported Savino et al. [36]. They found that the fasting ghrelin values were lower in the nursed babies than in formula-fed infants, however, the difference, was statistically insignificant in children over 4 month old [36].

One hour after the feeding, the concentrations of ghrelin in plasma were opposite to these in the fasting state. The ghrelin levels were the lowest in the children fed with highly hydrolyzed diet, and the highest level of this peptide was recorded in the breast-fed children. Although the energy intake in the ingested food was the same, the food differed in its composition, especially in the degree of hydrolysis of the proteins and carbohydrates and the level of ghrelin. According to Aydin et al. [37], ghrelin is present in the human milk and its concentration increases with the duration of lactation, however, the concentration of ghrelin in plasma is almost twice as high as in milk [37]. Taking into account the relatively high ratio of the volume of the nursed milk to the volume of infants blood, the higher levels of postprandial ghrelin in breast-fed children might result from exogenous delivery of this peptide from the mother milk. The impact of ingested (exogenous) peptides on the infants development has not been recognized yet.

An increase in the ghrelin levels after nursing might have an alternative explanation. The serum ghrelin concentrations increase on fasting and decrease after the meals [38]. However, decrease in postprandial ghrelin is not immediate. It can take up to 2 hours after meals [39,40]. In contrast to adults, ghrelin refractoriness to inhibition by food intake in children should reflect a peculiar functional profile of ghrelin in childhood [41]. The ghrelin in childhood is oriented to anabolic functions i. e. stimulation of the feeding behavior. The long-lasting high ghrelin levels after the meal in breast-fed children seem advantageous because it might support a development of so fed babies [42,43]. Additionally, the ghrelin plasma levels are related to maturation of the ghrelin production in the stomach. An increase in the ghrelin secretion during the first months of the life probably prepare the babies to differentiation of food, and specially to the solid food ingestion [43]. In this case the higher and longer lasting ghrelin levels - as observed in breast-fed babies - might be extremely beneficial.

The fasting OXA levels mimic the pattern of ghrelin. Plasma levels of orexins decrease with age and it could be related to the energy requirement (per kg body weight) [44]. The tested children received the same amount of energy and, therefore, differences of the OXA concentration might resulted from the disturbances in the hunger-satiety signaling, especially in the case of highly hydrolyzed diet.

The plasma levels of OXA one hour after the feeding were similar to these observed in the fasting state, except the breast-fed children showing postprandial increase in its concentration (p < 0.05, paired t-test), (figure 1). Plasma concentrations of OXA in adults increased on fasting and decreased after the meal [45]. The higher levels of OXA in the breast-fed children after the meal than prior to feeding might be considered in terms of the presence of OXA in human milk, but there is no evidence for secretion of orexins to the milk in humans. A specific stimulation of the gut with the human milk could offer another explanation, because orexin-positive neurons are localized not only in the hypothalamus, but also in the gastrointestinal tract [46]. A stimulation of OXA secretion with ghrelin could also be taken into account. Under such circumstances, the elevated postprandial ghrelin observed in breast-fed children levels would be responsible for a higher OXA output. However, in nursed babies and the cow milk formula-fed groups, the correlation between OXA and ghrelin was statistically insignificant.

Natural nursing is the gold standard in the infants' nutrition in the first months of their life. Therefore, all comparisons should be performed for the breast-fed babies. The present study revealed that the type of infant feeding influence concentrations of ghrelin and OXA in the plasma. Humanized cow milk is not equivalent to the human milk, but provides hardly the close resemblance. Therefore, it is not surprising that the ghrelin and OXA responses are similar to the physiological effect of the human milk ingestion. The significant differences in fasting and also postprandial plasma levels of OXA and ghrelin as compared to nursed children were observed in the children fed with highly hydrolyzed diet. In this group of children, the concentration of ghrelin negatively correlated with the glucose concentrations. All these data fitted well the observations that infants with small weight gain over the first year of their life have lower ghrelin levels after an intravenous glucose load [47]. Also Savino [35] found negative correlation between fasting plasma ghrelin concentrations and infant weight gain, suggesting that ghrelin concentration seems to be higher when infant growth is slower [35]. Thus, results of the present study support suggestions that children fed with the highly hydrolyzed formulas might face disturbed short-time development [48,49]. Prolonged application of the highly hydrolyzed formula might result in setting the basal and post stimulus levels of orexigenic peptides in points different from these essential for children fed with either human milk or cow milk formula. Long-term perspective studies would be required to elucidate real consequences of different diets applied.

In this group of children the concentration of ghrelin negatively correlated also with the concentrations of OXA. It is known, that secretion of OXA is controlled by ghrelin [22]. Likely, in the children on the highly hydrolyzed diet not only the secretion of ghrelin but also the relation between ghrelin and OXA is perturbed.

It should also be taken into account that the postprandial changes in the ghrelin and OXA secretion in highly-hydrolyzed formula (Nutramigen) -fed group might be developed faster than in other studied children. Nutramigen is based on highly hydrolyzed milk casein with addition of simple amino acids and glucose polymers. The digestion of such a mixture is fast, and, therefore, uptake of amino acids might elicit rapid stimulation of ghrelin secretion and faster decrease in the ghrelin plasma concentration [50]. Possible changes might be omitted in the "two-point blood sampling" study.

### Parenterally fed children

There are hardly few papers describing effects of the parenteral nutrition on hunger and satiation, and presented results are contradictory because of the complexity of the problem [51,52]. Several interfering factors such as age of the patients and its caloric needs, the primary illnesses, the duration of parenteral therapy *per se*, duration of infusion per day and, the most important, the ratio between parenteral and enteral energy delivery should be taken into account. In the course of this study, solely infants on complete parenteral provision of calories (total parenteral nutrition - TPN) were tested. The infusions were provided in around the clock schedule, to cover the calorie needs and decrease potential metabolic (acidosis) and clinic side effects (venous inflammation). Despite of differences in the calorie load there were no significant differences in the plasma levels of glucose, ghrelin and OXA in the children receiving solely glucose intravenous infusion and the children on total parenteral nutrition. However, the concentrations of the determined peptides were slightly higher in the TPN children, probably due to higher calorie provision and/or presence of amino acids and lipids in the infused fluids. Moreover, the concentrations of orexigenic peptides in these groups resembled the postprandial concentrations of ghrelin and OXA in the children fed with the cow milk formula. The observation that parenteral nutrition evoked changes in the orexigenic peptides such as oral bolus in patients on enteral nutrition agrees with the data in the sole paper on the subject published by Murray et al. [46] who analyzed ghrelin in parenterally fed humans. Data on OXA in parenterally fed humans are unavailable. In that experiment with adult patients, ghrelin levels markedly declined in course of either parenteral nutrition or dextrose infusions reaching values similar to the postprandial concentrations [52].

As shown in this study, the ghrelin and orexin plasma concentrations during the TPN mimics the postprandial levels observed in enterally fed children. A constant stimulation with no intrameal modulation of ghrelin and OXA secretion could be most harmful for children fed parenterally for a long time. As recorded by Bonifacio et al. [53], in the patients on long-term parenteral nutrition, BMI increased year by year [53]. This phenomenon might be related to the continuous stimulation of the secretion of orexigenic peptides and their anabolic properties, observed in the present study.

### Feeding in early life - long-term consequences - focus on obesity

Energy balance is efficiently regulated in the short- and long-term feedback. However, some differences in set-point of secretion of central and peripheral signals in the early stages of life might be crucial in the weight outcome in the further life. The neonatal and infant period might be considered as a new 'orexigenic' condition setting the point or providing programming of ghrelin and even the whole orexigenic regulation. The concept that nutrition in the infancy could have a long-term influence on adiposity and even program it was formulated in the 60'ties of the past century [54]. The beginning of the enteral feeding determines consequences for the regulation of orexigenic (and anorexigenic) factors, especially, when it is superimposed with the type of the feeding. This idea - very difficult to prove - is supported by several facts. It has been shown that maternal prepregnant body mass index, the duration of breastfeeding, and the timing of complementary food introduction are associated with the infant weight gain from the birth to the first year of life. Also healthy infants with higher cord leptin levels at birth gain weight more slowly over the period from their birth up to 4 months [55,56]. The influence of early nutrition on a long-term adiposity has already been shown [51]. It focused on the possible protective role of the breastfeeding and the differences in the development between breast-fed and formula-fed infants [57].

Present study showed that these disturbances involve not only fasting plasma ghrelin levels but also plasma concentration of OXA and postprandial secretion of ghrelin and OXA. Observed discrepancies might be related to some longitudinal effects upon the development or disease in the further life. It is well documented by meta-analysis that breast-fed children have a lower risk of elevated weight gain in the further life [58-62], Some studies suggested a longitudinal effect of nutrition in early life on the adult atherosclerosis [63,64]. The hypothesis on the adiposity programming with the type of feeding based mainly on chemical constitution of the human milk and absence of some bioactive nutrients in formulas [65]. These specific components either set points or programme of the basal/fasting and postprandial concentration or secretion of hormones and bioactive peptides. As shown in this study, there was a significant difference in fasting and postprandial ghrelin and OXA levels in differently fed infants. It could be hypothesized that the feeding in the early live set the point of gut-brain orexigenic axis and that it was responsible for several longitudinal effects indicated above. Taking into account the ghrelin and OXA concentrations observed in this study in breast-fed and formula-fed infants, in our opinion the cow milk formula slightly affected the orexigenic axis. The infants fed with the highly hydrolyzed formula the most distinguished from the other groups in the secretion of peptides involved in this study, even when the group with the parenteral nutrition was included in the comparison. High fasting concentration of ghrelin might be associated with a stronger feeling of hunger. The fixed tendency to higher fasting ghrelin levels should provide a greater risk of obesity in further life. The low postprandial concentration of ghrelin related, probably, to a fast down-regulation of the post-stimulus secretion also might lead to obesity. Therefore, the improper secretion of these two anabolic peptides should be taken into account as possible factors inducing disturbances in development.

## Conclusion

The highly hydrolyzed diet strongly affects fasting and postprandial ghrelin and orexin plasma concentrations with possible negative effect upon short- and long-time effects in the development. The continuous stimulation and lack of fasting/postprandial modulation of ghrelin and orexin concentrations might be responsible for disturbed development of parenterally fed children.

## Competing interests

The authors declare that they have no competing interests.

## Authors' contributions

The authors' contribution to the present paper was equal. Authors read and approved the final manuscript.

## Pre-publication history

The pre-publication history for this paper can be accessed here:

http://www.biomedcentral.com/1471-230X/9/92/prepub
